# Remotely-Sensed Indicators of N-Related Biomass Allocation in *Schoenoplectus acutus*

**DOI:** 10.1371/journal.pone.0090870

**Published:** 2014-03-10

**Authors:** Jessica L. O’Connell, Kristin B. Byrd, Maggi Kelly

**Affiliations:** 1 Department of Environmental Sciences, Policy and Management, University of California, Berkeley, Berkeley, California, United States of America; 2 United States Geological Survey, Menlo Park, California, United States of America; Dauphin Island Sea Lab, United States of America

## Abstract

Coastal marshes depend on belowground biomass of roots and rhizomes to contribute to peat and soil organic carbon, accrete soil and alleviate flooding as sea level rises. For nutrient-limited plants, eutrophication has either reduced or stimulated belowground biomass depending on plant biomass allocation response to fertilization. Within a freshwater wetland impoundment receiving minimal sediments, we used experimental plots to explore growth models for a common freshwater macrophyte, *Schoenoplectus acutus*. We used N-addition and control plots (4 each) to test whether remotely sensed vegetation indices could predict leaf N concentration, root:shoot ratios and belowground biomass of *S. acutus*. Following 5 months of summer growth, we harvested whole plants, measured leaf N and total plant biomass of all above and belowground vegetation. Prior to harvest, we simulated measurement of plant spectral reflectance over 164 hyperspectral Hyperion satellite bands (350–2500 nm) with a portable spectroradiometer. N-addition did not alter whole plant, but reduced belowground biomass 36% and increased aboveground biomass 71%. We correlated leaf N concentration with known N-related spectral regions using all possible normalized difference (ND), simple band ratio (SR) and first order derivative ND (FDN) and SR (FDS) vegetation indices. FDN_1235, 549_ was most strongly correlated with leaf N concentration and also was related to belowground biomass, the first demonstration of spectral indices and belowground biomass relationships. While *S. acutus* exhibited balanced growth (reduced root:shoot ratio with respect to nutrient addition), our methods also might relate N-enrichment to biomass point estimates for plants with isometric root growth. For isometric growth, foliar N indices will scale equivalently with above and belowground biomass. Leaf N vegetation indices should aid in scaling-up field estimates of biomass and assist regional monitoring.

## Introduction

Belowground biomass of wetland emergent plants is the dominant source of soil organic carbon and peat in wetlands [1], [2] and contributes to long-term carbon sequestration [3]. In addition to soil organic carbon storage, wetland belowground biomass also contributes to soil stability and accretion by adding soil organic matter volume [4], [5]. Many coastal wetlands may subside below sea level because of a combination of surface water drainage, salt water intrusion, marsh compaction (peat collapse), sediment starvation, and global sea level rise [6]–[11]. As wetlands subside, they become excessively inundated, resulting in flooding, plant death, and marsh loss. Wetland loss can be problematic because wetlands provide many functions and services to society, such as flood water mitigation, storm surge attenuation, belowground carbon storage, water filtration and purification, and habitat for wetland biota [12]. Belowground biomass may consequently maintain or build wetland elevation to prevent subsidence below sea level [13]. Therefore, tools for predicting and promoting wetland plant belowground biomass can support wetland management for resilience and provision of ecosystem benefits.

Two competing hypotheses explain relationships between below and aboveground biomass. The balanced-growth hypothesis is an ecological model which suggests, all else being equal, plants allocate growth towards the most limiting resource, e.g. towards shoots when light is limited, or towards roots when nutrients or water are limited [14], [15]. This ecological concept also has been called optimal partitioning theory [16]. Alternatively, above and belowground biomass ratios may conform with the isometric allocation model, which suggests that above and belowground biomass scale equivalently across multiple environmental conditions [16]. Plant growth can correspond to a balanced-growth model [17]–[19] or an isometric allocation model [20], [21]. In both situations, leaf N concentration can inform belowground biomass estimates. In the balanced growth case, additional nutrients, such as N and P, increase shoot growth but do not increase belowground biomass equally, therefore leaf N scales with root:shoot ratios. Alternatively, plants responding isometrically to nutrient addition will have stimulated below and aboveground biomass and constant root:shoot ratios.

While much progress has been made estimating aboveground biomass from optical remote-sensing technology [22], [23], monitoring belowground biomass remains challenging. Belowground biomass measures are difficult because biomass ratios in plants sometimes vary with access to nutrients, light, competition, temperature, and phylogenetic history [14]. As a result, while above and belowground biomass usually are correlated, sometimes root:shoot ratios fluctuate greatly across vegetation types and environments [24].

Past studies indicate the potential for remote sensing to detect eutrophic conditions. In particular, remote sensing has been commonly used to estimate foliar nitrogen concentration in aboveground plant tissue [22], [25]–[31]. For nutrient limited species, plants grown in high N environments may concentrate N in aboveground tissues at greater levels than otherwise [32], [33]. When combined with appropriate growth models (balanced or isometric growth), leaf N might also provide evidence of belowground biomass when species specific field data are available to calibrate site models.

Several analytical techniques have been developed to relate hyperspectral data to canopy N concentration, such as band-depth analysis [34], spectral matching techniques [35], and partial least squares regression [22]. However vegetation indices correlated to plant characteristics through empirical methods, are a simple, straightforward and rapid method for detecting the biochemical absorption signal while minimizing background effects [36]–[38]. Therefore, we consider the use of vegetation indices derived from freely-available Hyperion hyperspectral imagery as a potential tool for land management. Hyperion is a hyperspectral satellite sensor on the National Aeronautics and Space Administration Earth Observing One Satellite (NASA EO-1), and has been successfully applied to map plant biophysical and biochemical characteristics [30], [39], [40].

**Figure 1:**
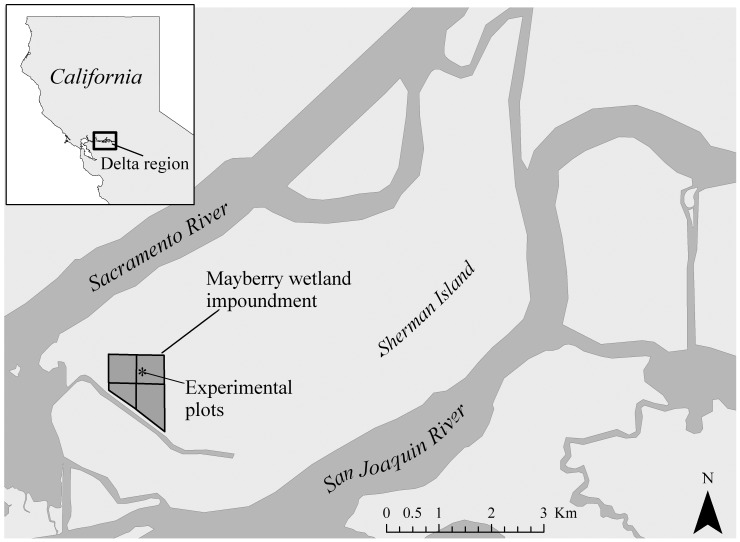
Study site. The Sacramento-San Joaquin River confluence and associated Delta within California’s Central Valley, USA. Locations of the Mayberry wetland impoundment and experimental site are indicated.

Here we explore plant growth models in a novel conservation context: freshwater peat marshes within California’s Sacramento-San Joaquin Delta region (Figure 1). The Delta is a wetland complex responsible for collecting a vast quantity of water for human use. Historically, the Delta was a 1400-km^2^ tidal marsh network [41] and contained a large area of emergent marshes with peat 2–15 m deep, a substantial carbon pool [42]. Wetland dewatering, peat decomposition, soil compaction and crop exploitation resulted in land subsidence of up to 8 m throughout the Delta [42].

We use an experimental approach to estimate biomass allocation of *Schoenoplectus acutus*, hardstem bulrush, an emergent macrophyte common to the Delta, and to freshwater marshes throughout North America [43]. We use our experimental model to draw relationships across simulated eutrophication (N addition), field measurements of whole plant biomass production, and remotely-sensed vegetation indices of leaf nitrogen concentration. Our main questions include 1) Can percent total N concentration in leaves be used to estimate belowground biomass? and 2) Do remotely-sensed vegetation indices indicate belowground plant response to eutrophic conditions? Vegetation indices may help identify eutrophic wetlands and assist with monitoring wetland nutrient cycling. Relationships between leaf N and belowground biomass might assist estimation of spatial variation in roots and rhizomes across and within sites, supporting regional carbon monitoring efforts. Such estimates also may identify areas where marsh subsidence or accretion are likely across broad areas.

## Methods

We installed 18 plant growth chambers in the northeast unit of an impounded wetland north of Mayberry Slough on Sherman island, CA, USA (Lat: 38.0533 Long: −121.7682). The Mayberry wetland is 122 ha of restored freshwater emergent marsh with 4 impounded units (Figure 1). Water levels within this wetland are managed by the California Department of Water Resources. Water flows from south to north from Mayberry Slough through the impoundments via water control structures.

Growth chambers were constructed by tacking landscaping cloth over a cylindrical frame constructed of bamboo (diameter: 30.5 cm, height: 135 cm). Growth chambers allowed water exchange with the surrounding wetland. Growth chambers were installed within the impoundment pond, 1 m from the impoundment shoreline, on 28 May 2012. Differences in pond bottom geomorphology resulted in differences (±15 cm) in water depth surrounding growth chambers. Each growth chamber was filled with 45 L of planting media (sphagnum peat moss), raising the effective water depth. Within each growth chamber, we planted 1-year-old *S. acutus* (from California Flora Nursery, Fulton, CA, USA, www.calfloranursery.com; propagules collected from the Russian River watershed, CA, USA, 140 km northwest of Mayberry Slough). We used growth chambers to allow accurate measurement of whole plant biomass. Growth chambers facilitate extraction of the entire root and rhizome system.

Plants were randomly assigned to control or N-addition treatments and installed in growth chambers. Growth chambers for these treatments were physically separated by a small incomplete levy, but open to the larger impoundment. Control treatment plants were also located upstream of the N-addition treatment in the wetland impoundment. This design ensured both treatments were similar hydrologically, except that the control treatment did not receive N additions. Within each chamber, we measured number of stems, stem height, stem diameter, and number of inflorescences monthly. The growth form of *S. acutus* is graminoid, consisting of one long cylindrical stem terminating in an inflorescence (when present) [43]. Therefore stem and inflorescence measurements adequately capture aboveground growth. We also measured water depth inside and outside of growth chambers.

Plants within the N-addition treatment were fertilized with custom blend controlled release fertilizer (Apex, 16-10-13, Simplot Professional Products Lathrop, CA, USA, apexfertilizer.com). Fertilizer was coated granules, designed for controlled nutrient release without influence from media type, moisture level, pH, or microbial activity. We added 50 g of Apex on 28 May 2012 and again on 2 July 2012. In addition, 5.65 g of uncoated granular fertilizer (Rootblast, 2-1-2, Rootblast international, Canton, Ohio, USA) were added on 28 May, 11 July, 2 August, and 25 September 2012. In total, 16.5 g inorganic N as nitrate (NO_3_), 4.5 g P as phosphate (PO_4_^-3^) and 13.5 g K as potash (K_2_O) were added. This represents a 4∶1 ratio of N:P by weight, as observed in the most eutrophic conditions of San Francisco Bay Estuary [44]. A fertilizer blend of NPK was added because increases in these nutrients commonly co-occur in eutrophic estuaries [44], [45]. Nutrient additions were added monthly because nutrient pulses (as opposed to continuous high concentrations) are common in natural conditions, where nutrients fluctuate temporally [32], [46], [47].

Crawfish (*Pacifastacus*, *Procambarus* or *Orconectes* sp.) [48] colonized 4 growth chambers per treatment on 2 August 2012. Entry holes created by crawfish allowed planting media to escape, flooding plants and causing mortality. We harvested all other plants on 1 October 2012 (N = 4 per treatment). All subsequent analyses were focused on these harvested plants. Plants were sorted into aboveground biomass (stems, leaves and inflorescences) and belowground biomass (roots and rhizomes). We did not separate live from dead roots because dead roots were fine roots. We could not reliably determine the transition from live to dead fine roots. All plant biomass was oven dried at 40°C until all plant tissue water was removed. Subsequent analyses focused on oven-dried weights. For simplicity, we defined root:shoot ratio as the ratio of all harvested above and belowground biomass.

### Estimates of Plant Biophysical and Biochemical Parameters

We used end of season above and belowground biomass as our biomass estimates. Oven-dried plant leaves were measured for total N (percent by weight) at the UC Davis analytical laboratory (Davis, California, USA; http://anlab.ucdavis.edu/). The Davis laboratory uses sample combustion coupled with thermal conductivity/IR detection (LECO FP-528 and TruSpec CN Analyzers) to measure total N. We estimated total N content (g) in leaves as: % leaf total N*aboveground biomass (g).

We used permutation methods to assess one-sided hypotheses, specifically that N addition increased whole plant and aboveground biomass, number of inflorescences, leaf N concentration (%) and leaf N (g), but reduced belowground biomass (total belowground biomass, and roots and rhizomes separately) and also reduced root:shoot ratios. We used all possible permutations (the COIN package in R, version 2.15.1, The R Foundation for Statistical Computing). Permutation tests are useful because their only assumption is that data are independent [49]. Permutation methods compute the probability that two samples come from the same population by generating a reference distribution for many permutations of the data [49]. We further evaluated whether leaf N concentration had significant relationships with root:shoot ratios using 10,000 bootstrap replicates with bias corrected, accelerated confidence intervals for linear models in program R.

### Vegetation Indices of Foliar Nitrogen Concentration

Canopy reflectance spectra were obtained over each plot near midday on 25 September 2012 (6 days before plant harvest) using an ASD Inc. FieldSpec Pro FR portable spectroradiometer (Analytical Spectral Devices, Inc., Boulder, CO, USA). Spectral readings were sampled every 1.4 nm over 350–1000 nm and 2 nm over 1000–2500 nm using a 25° field of view foreoptics. Readings were taken at nadir 1 m above the vegetation canopy using a 3-m optical fiber cable. Canopy reflectance was calculated as the ratio of canopy radiance to radiance measured from a calibrated white reference, Spectralon (Labsphere, Inc.), which was collected every 10 min. At each sample location, ten reflectance measurements, each an average of 12 spectra, were collected and then averaged using ViewSpec Pro 6.1.10 (Analytical Spectral Devices, Inc, Boulder, CO, USA).

Leaf N concentration can be estimated from spectral indices such as normalized difference indices (ND, equation 1) or simple band ratios (SR, equation 2) [28]. In addition, NDs and SRs calculated from first-order derivatives of the reflectance spectrum have also been related to N concentration [25], [50], [51]. First-order derivatives, calculated in ViewSpec Pro 6.1.10 [52] involve the calculation of the slope of the spectrum. First derivative spectra are useful for reducing the effects of multiple scattering of radiation due to sample geometry and surface roughness [53] and for enhancing absorption features and inflection points masked by interfering background absorptions and canopy background effects [54]–[56]. Given the background absorptions of the surrounding water, we expected that indices derived from first derivative spectra would perform well in estimating leaf nitrogen concentration.
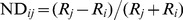
(1)

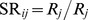
(2)where *R_i_* and *R_j_* are the reflectance values of bands *i* and *j*.

ND or SR indices using new band combinations may be more highly correlated to leaf biochemical properties than those more commonly found in the literature [57]. As a result, we tested multiple published indices (see Table 1) and new indices to determine which were most related to leaf N concentration in our experimental plots. To test new hyperspectral indices we first simulated 164 hyperspectral Hyperion bands with the field spectroradiometer data. Hyperion has a 30-m spatial resolution and 242 bands each 10 nm-wide. The simulated Hyperion bands covered the ranges 422–1300 nm, 1443–1795 nm, and 1998–2400 nm. Water absorption regions of the spectrum with high noise were removed from analysis. These field-collected simulated Hyperion bands represent finely detailed full-spectrum data and provide a point of comparison for future field studies using other remote sensing platforms.

**Table 1:** Previously identified relationships between leaf N concentration and hyperspectral vegetation indices.

Published N Indices
FDS_743,1316_ [25]
ND_483,503_ [66]
SR_805,710_ [77]
SR_800,680_ [51]
SR_700,550_ [78]
SR_810,560_ [79]
REP [80]
NDNI [36]

REP (red edge position index) = 700+40(R_RE_−R_700_)/(R_740_−R_700_), R_RE_ = (R_670_+R_780_)/2.

NDNI (Normalized Difference Nitrogen Index) = [log(1/R_1510_)−log(1/R_1680_)]/[log(1/R_1510_)+log(1/R_1680_)].

For the simulated dataset, we calculated all possible ND and SR indices using all band combinations [57] for a total of 13,366 Hyperion indices for each index type. We also calculated the first order derivative spectra in ViewSpec Pro 6.1.10, and calculated all possible first derivative NDs (FDN) and first derivative SRs (FDS) using all band combinations for a total of 13,366 indices for each index type.

Using Stata/SE 12.1 (StataCorp LP 1985–2011), we ran pairwise correlations to calculate the correlation (*r*) between all indices (published and new) and leaf N concentration. We used leaf N concentration (%) rather than leaf N (g) because the latter is confounding with aboveground biomass (i.e. as aboveground biomass increases, total weight of N increases concomitantly, but % N per unit leaf might not increase) [28]. Coefficients of determination (*R* ^2^) were calculated as r*r. We also compared the bands selected in these high *R^2^* indices against known N absorption bands to evaluate the physical basis for index selection [36], [58], [59]. We used 10,000 bootstrap replicates with bias corrected, accelerated confidence intervals for linear models in program R to investigate how well the best indices were related to belowground biomass and root:shoot ratio. We present results for those indices whose correlations with leaf N were greater than 80% and whose bootstrapped 95% CI’s for slope in regressions with leaf N and belowground biomass did not overlap zero. Given our small sample size, we consider this latter criterion to be conservative and more reliable than p-value for estimating statistical significance.

### Ethics Statement

Permission was given for site access by the California Department of Water Resources. No special permits were required for our research sampling scheme.

## Results

### Plant Biophysical Parameters

Whole plant biomass (belowground+aboveground) was similar between treatments (Table 2), though aboveground biomass was 71% greater in the N-addition than in the control treatment (Table 2). Conversely, belowground biomass was 36% greater in the control than in the N-addition treatment (Table 2). This resulted in a root:shoot ratio 74% greater in the control than in the N-addition treatment (Table 2). Inflorescence production also was higher in N-addition plots than in control plots (Table 2). Further, leaf N concentration was 18% and leaf N (g) was 77% greater in the N-addition treatment than in the control (Table 2). Finally, leaf N concentration had a significant negative relationship with root:shoot ratio in *S. acutus* (Table 3). We did not detect a significant relationship between below and aboveground biomass (Table 3).

**Table 2:** Differences between *Schoenoplectus acutus* biophysical parameters within N-addition and control treatments based on permutation tests (N = 4 per treatment).

Response variable	*Z*	*P*	Control mean ± SE	N-addition mean ± SE
Whole plant biomass (g)	1.03	0.85	133.4±61.3	112.2±23.1
AG biomass (g)	−2.02	**0.02**	**9.6±2.7**	**32.9±7.6**
BG biomass (g)	1.34	*0.09*	*123.8±58.9*	*79.3±19*
Roots: rhizomes	−0.29	0.4	1.9±0.5	1.6±0.2
Roots:shoots	2.08	**0.02**	**10.4±3.3**	**2.7±0.6**
Inflorescence number	−1.13	*0.07*	*0*	*3±2.3*
Leaf N concentration	−1.60	*0.09*	*1.4±0.1*	*1.7±0.2*
Leaf N (g)	−1.68	**0.03**	**12.8±2.7**	**56.4±16.4**

*P<0.10*; ***P*** **<0.05**;

BG = belowground; AG = aboveground.

**Table 3:** Relationship of plant biophysical parameters with potential biophysical, chemical, and remote sensing-based nitrogen predictors (N = 8); 95% CI is the bootstrap bias corrected, accelerated 95% confidence interval based on 10,000 bootstrap replicates.

Model	95% CI Intercept	95% CI Slope	*Adj-R^2^*	*F*	*P*
BG biomass = AG biomass	−34.2–327.9	−12.0–8.8	0.05	0.3	0.63
Root:shoot ratio = Leaf N	**6.0–53.3**	−**32.8–**−**1.7**	**0.49**	**7.8**	**0.03**
BG biomass = FDN_1235, 549_	*79.6–485.2*	*11.4–*−*1326.4*	*0.33*	*4.4*	*0.08*
Root:shoot = FDN_1235, 549_	*3.6–29.9*	*0.1–76.3*	*0.27*	*3.6*	*0.1*
AG biomass = FDN_1235, 549_	−42.5–98.5	−164.6–115.1	−0.14	0.2	0.68
Leaf N = FDN_1235, 549_	**0.6–1.1**	−**3.1–**−**1.4**	**0.89**	**58.1**	**<0.01**
BG biomass = FDS_2184, 1780_	−**39.1–103.5**	−**239.6–**−**8.6**	**0.44**	**6.5**	**0.04**
Root:shoot = FDS_2184, 1780_	−**4.2–5.9**	−**18.0–**−**1.2**	**0.54**	**9.3**	**0.02**
AG biomass = FDS_2184, 1780_	7.5–53.7	−20.6–45.5	−0.01	0.9	0.37
Leaf N = FDS_2184, 1780_	**2.4–3.2**	−**3.1–**−**1.7**	**0.88**	**53.1**	**<0.01**

*P<0.10*; ***P*** **<0.05**;

FDS = first derivative simple ratio index;

FDN = first derivative normalized difference index;

BG = belowground; AG = aboveground.

### Vegetation Indices for Foliar Nitrogen Concentration

**Figure 2:**
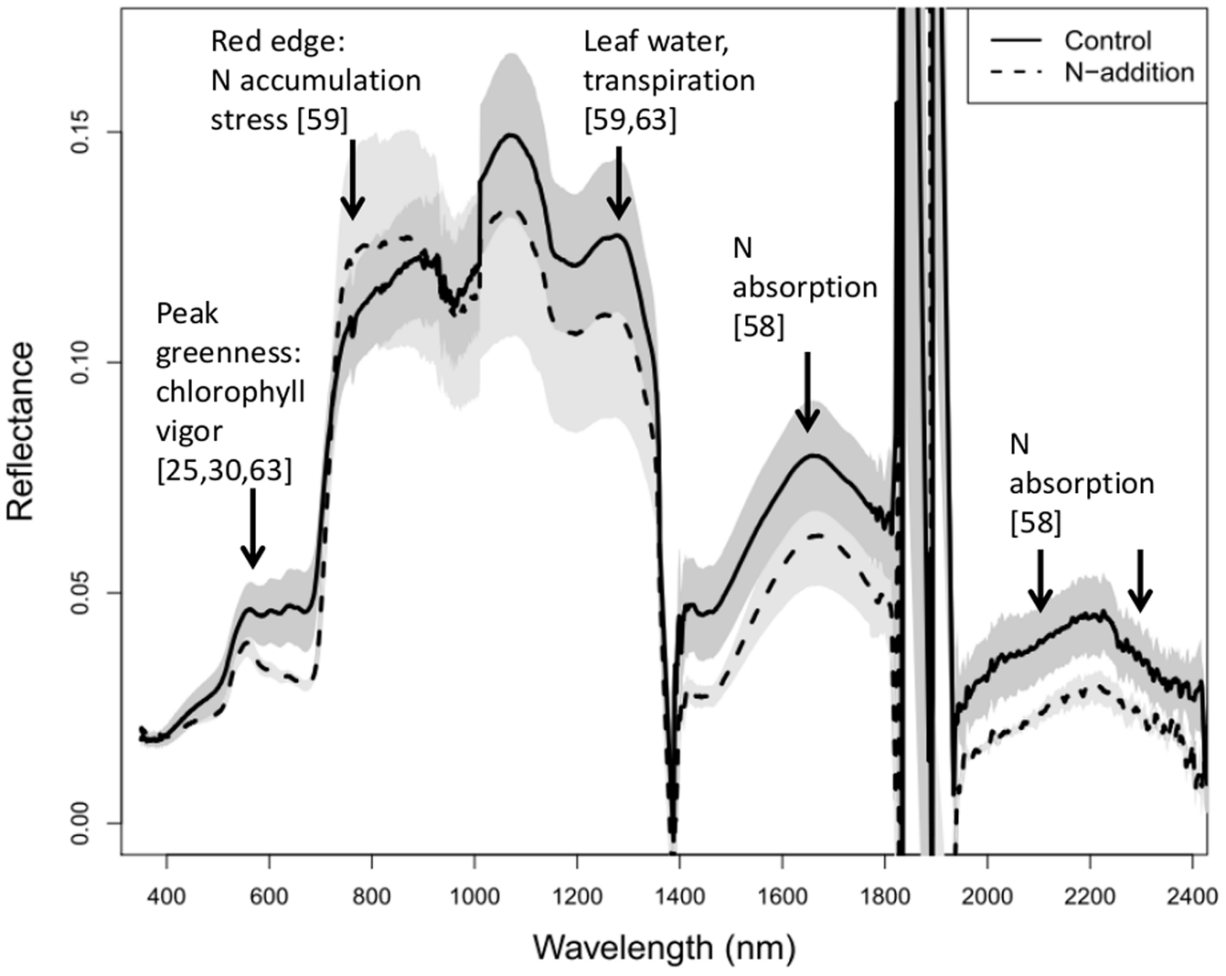
Reflectance spectra for treatments. Averaged reflectance spectrum for N-addition and control plants (N = 4 each). The shaded grey areas represent ± one standard error. Spectra associated with foliar N in the literature are indicated, with corresponding citations in brackets.

Most published indices (Table 1) were poorly correlated with leaf N concentration, with the exception of FDS_743,1316_ (*F*_1,6_ = 5.38, *R* ^2^ = 0.47, *P = *0.06). Among all indices generated from simulated Hyperion bands, FDN_1235,549_ was most correlated with leaf N concentration (Table 4). The use of green wavelengths (∼500 to 570 nm) in hyperspectral indices have been found to be sensitive to N and can improve the precision and accuracy of predictions [28]. In our experiment, green indices based on spectral reflectance were not significant, though green indices using first-order derivatives of reflectance were highly significant. The difference in the green peak region between treatments is evident when comparing the average reflectance spectrum of N-addition plots to those in control plots (Figure 2). Of the other best new indices, most contain bands in the short-wave infrared region of the spectrum (Table 4). Finally, two of the best indices explaining leaf N concentration (FDN_1235,549_ and FDS_2184,1780_) also exhibited a positive relationship with belowground biomass and root:shoot ratio, but were unrelated to aboveground biomass (Table 3). All other top indices for leaf N (correlation>0.8) were also related to belowground biomass, (*P*<0.05) but 95% CI’s for slope overlapped zero. Therefore, we did not present these.

**Table 4:** Best new indices for each index type.

N	midpoint	midpoint	
Indices	Band 1	Band 2	*R* ^2^
FDN	1296	508	0.795
	1296	681	0.796
	2002	1155	0.822
	2184	1780	0.846
	1235	549	0.903
FDS	2194	1124	0.871
	2194	1235	0.877
	2194	1033	0.894
	1235	549	0.895
	2194	752	0.897
ND	1245	1205	0.685
	2274	2264	0.694
	1245	1215	0.713
	1235	1225	0.764
	2264	2244	0.793
SR	1245	1205	0.684
	2274	2264	0.692
	1245	1215	0.713
	1235	1225	0.764
	2264	2244	0.792

FDS = first derivative simple ratio index;

ND = normalized difference index;

SR = simple ratio index of;

FDN = first derivative normalized difference index.

## Discussion

Whole plant biomass was equivalent among treatments, but above and belowground biomass did not scale equivalently as predicted under isometric growth models. Further, simulated eutrophication (N-addition) directed plant growth away from roots and rhizomes and towards leaves and inflorescences. Plants may increase belowground biomass in low nutrient conditions both to increase nutrient absorptive area and to increase root storage of carbohydrates to buffer against future scarcity [60]. In our experiment, both were observed as control plots invested more in belowground biomass, while root:rhizome ratios did not differ between treatments. Thus, plants in the N-addition treatment maximized energy capture and reproduction potential but reduced their contribution towards generating stable belowground organic matter that could contribute to wetland accretion [5], [13] and peat formation [2]. Our results suggest *S. acutus* conforms to the balanced-growth model in northern California wetlands, as has been observed for other plant species elsewhere [15], [17]–[19], [60]. Plants do not always exhibit balanced growth [16], [20], therefore further investigations are necessary for individual species under specific growing conditions. Where plants grow isometrically, vegetation indices of foliar N may add information to aboveground biomass measures by indicating where N-enrichment increases whole plant biomass production. In our study, simulated eutrophication was detectable by vegetation indices of leaf nitrogen concentration. Further vegetation indices were related to end of season root:shoot ratio and belowground biomass in *S. acutus*. Relationships between leaf N and vegetation indices generally are species, season and site specific [28], [61]. Differences in species composition, plant structure and substrate will require re-estimation of these relationships elsewhere.

Vegetation indices detected increased foliar N concentration in *S. acutus* resulting from N addition, as noted in other wetlands [37]. Plants take up nutrients through their roots, and can use them for immediate growth or to store excess nutrients for future needs [32], [33], [60]. Plants may increase N content by both increasing N per tissue unit or by increasing the number and length of shoots, leaves and inflorescences. Both mechanisms were observed here. Plant N concentration often is a reliable bioindicator of water quality because watersheds experience nutrient pulses, while plants integrate average conditions by absorbing nutrients from water and sediments over time [37], [46], [47], [62].

In a data mining approach such as used in this study, there is the possibility of finding spurious correlations. Spurious correlations might be observed when multispecies responses are not accounted for, or when reflectance due to species differences in canopy architecture, differences in background effects, or scattering from leaf surface features obscure reflectance from leaf interior components, such as foliar nitrogen [61]. As a result final index selection should have a physical or biological basis [38], [61]. First derivative indices were more closely related to nitrogen concentration than reflectance indices. Similar to results in Townsend et al. [30], the use of first-derivative spectra helped identify features related to foliar nutrient concentration. Bands with midpoints at 549 and 752 nm represent the slope at peak greenness and the red edge, respectively. While there are no absorption features for nitrogen in chemical bonds at shorter wavelengths, reflectance at 550 nm is related to chlorophyll concentration, and serves as a surrogate indicator of N levels and plant vigor [25], [30], [63]. Reflectance at the red edge is related to nitrogen accumulation and plant stress [59]. Nitrogen occurs primarily in proteins and chlorophylls of leaves, and because the two variables are moderately correlated within and across ecosystems, many researchers have associated the spectroscopic estimation of nitrogen to that of chlorophyll pigments [26]. Likewise reflectance at wavelength 1235 nm is near an absorption peak related to leaf water content [59], [63]. Leaf water content is indicative of plant vigor because of leaf water relationships with plant turgor, thermoregulation and photosynthesis [26].

The first derivative spectrum is used to identify absorption features through relative rate of change of reflectance within a wavelength region, and bands related to N concentration may be adjacent to known absorption features instead of centered on these features [30]. Other top indices (Table 4) are based on bands in the shortwave infrared region near to nitrogen absorption regions of 1730, 2180, 2240 [58]. Bands past 2300 were not considered due to noise in the spectrum.

Vegetation indices of leaf N had significant relationships with root:shoot ratios and belowground biomass in *S. acutus* and might be used to identify relative differences in belowground biomass. Our results are firmly grounded in past research, which has shown that optical remote sensing can be used to estimate leaf N concentration [25]–[30], [39], [64]–[66], that nitrogen inputs often increase leaf N [67]–[70] and sometimes concomitantly reduce belowground biomass [17]–[19]. In isometric growth, N enrichment may stimulate above and belowground productivity equivalently [20], [21], [71], [72]. Our study is the first to make use of remotely-sensed signals of whole-plant nitrogen response to link growth models and belowground biomass estimates.

### Applications to Wetland Conservation and Monitoring

Our analytical approach involved single species experimental data. Our conclusions provide evidence that nutrient-enrichment may direct biomass allocation for *S. acutus* and can be detected by spectral reflectance signals. Applying our approach in a field setting will require overcoming additional challenges. For example, we held confounding variables equal among treatments. This allows identification of the magnitude of biomass allocation shifts from nutrient addition alone. In field settings, hydrology related parameters such as water depth, dissolved oxygen and nitrogen will co-vary [73]. Therefore field-measured vegetation indices of foliar N should represent the sum of several environmental conditions and will require further study.

An additional challenge is in addressing differential species responses to N-enrichment in diverse communities. Species invariate spectral reflectance modeling of canopy N may be unreliable [61]. To avoid this problem, researchers might map sites according to dominant cover types and explore species specific models within these. In wetlands, such an approach may be particularly useful. Wetlands have strong hydrology related environmental gradients and wetland species have specific adaptions to stress, causing species sorting. Examples include salt marshes dominated by monospecific stands of *Spartina alterniflora*, brackish marshes dominated by *Spartina patens*, or freshwater marshes dominated by several cover types that occur in distinct patches. Sites with high spatial heterogeneity in species composition may not be appropriate for our approach, but a field study is needed to substantiate this.

Estimates of belowground biomass under varying nutrient levels will assist with conservation management of freshwater marshes experiencing subsidence. In coastal marshes, decreases in belowground biomass contribute to soil instability, compaction, erosion, and marsh loss [18], [19]. Coastal marshes experience frequent disturbances, such as strong storms and hurricanes, which might accelerate habitat loss where low belowground biomass results in soil instability [74]. Vegetation indices of leaf nitrogen might be used to identify areas where root growth is relatively low and therefore vulnerable to storm damage across broad regions.

Further, the Sacramento-San Joaquin River Delta region of California is experiencing large subsidence below sea level on marsh lands converted to agriculture [42]. Numerous marsh restoration projects have been proposed or are in progress across the Delta (California Delta Initiative; http://www.water.ca.gov/deltainit). Belowground biomass production within the Delta contributes to wetland accretion of 3–9 cm yr^-1^ within restored freshwater wetlands of mixed *S. acutus* and *Typha* sp. [5]. Vegetation indices should be useful for indicating spatial patterns of both low and high root growth in *S. acutus* and suggest appropriate management to meet target restoration goals. We are currently testing these indices in field settings dominated by *S. acutus* and *Typha* species in the Sacramento-San Joaquin Delta.

Estimates of belowground biomass in marshes are useful for following nutrient and carbon cycling. For example, wetlands frequently are used to treat reclaimed N-rich wastewater. Vegetation indices can help monitor canopy N concentration both spatially and temporally [28], [29]. Further, belowground biomass contributes to peat formation and stable soil organic carbon complexes [2], [5], [75]. Estimates of belowground biomass therefore are useful for global carbon cycling models [76]. Vegetation indices such as these can be applied across broad areas and contribute to our understanding of vegetation dynamics.

## Supporting Information

Dataset S1 **First derivative reflectance data used in this study.** Abbreviations are as follows: plot is an arbitrary number indexing samples; p_nitrogen is percent foliar N, treatment is treatment with values of N reflecting nitrogen addition and C the control; AB and BG biomass are aboveground and belowground biomass in grams; wXXX (e.g. w350–w2500) are the first derivative of reflectance at wavelength XXX, where wavelength is measured in nanometers.
(ZIP)
